# Metabolomic analyses reveal substances that contribute to the increased freezing tolerance of alfalfa (*Medicago sativa* L.) after continuous water deficit

**DOI:** 10.1186/s12870-019-2233-9

**Published:** 2020-01-08

**Authors:** Hongyu Xu, Zhenyi Li, Zongyong Tong, Feng He, Xianglin Li

**Affiliations:** 1 Institute of Animal Science, Chinese Academy of Agricultural Sciences, Beijing, People’s Republic of China; 2 College of Grassland Science, Qingdao Agricultural University, Qingdao, People’s Republic of China

**Keywords:** Freezing tolerance, Alfalfa, Forage grass, Water deficit, Metabolomics, LC-MS

## Abstract

**Background:**

Alfalfa is a high-quality forage cultivated widely in northern China. Recently, the failure of alfalfa plants to survive the winter has caused substantial economic losses. Water management has attracted considerable attention as a method for the potential improvement of winter survival. The aim of this study was to determine whether and how changes in the water regime affect the freezing tolerance of alfalfa.

**Results:**

The alfalfa variety WL353LH was cultivated under water regimes of 80 and 25% of water-holding capacity, and all the plants were subjected to low temperatures at 4/0 °C (light/dark) and then − 2/− 6 °C (light/dark). The semi-lethal temperatures were lower for water-stressed than well-watered alfalfa. The pool sizes of total soluble sugars, total amino acids, and proline changed substantially under water-deficit and low-temperature conditions. Metabolomics analyses revealed 72 subclasses of differential metabolites, among which lipid and lipid-like molecules (e.g., fatty acids, unsaturated fatty acids, and glycerophospholipids) and amino acids, peptides, and analogues (e.g., proline betaine) were upregulated under water-deficit conditions. Some carbohydrates (e.g., D-maltose and raffinose) and flavonoids were also upregulated at low temperatures. Finally, Kyoto Encyclopedia of Genes and Genomes analyses revealed 18 significantly enriched pathways involved in the biosynthesis and metabolism of carbohydrates, unsaturated fatty acids, amino acids, and glycerophospholipids.

**Conclusions:**

Water deficit significantly enhanced the alfalfa’ freezing tolerance, and this was correlated with increased soluble sugar, amino acid, and lipid and lipid-like molecule contents. These substances are involved in osmotic regulation, cryoprotection, and the synthesis, fluidity, and stability of the cellular membrane. Our study provides a reference for improving alfalfa’ winter survival through water management.

## Background

Alfalfa (*Medicago sativa* L.) is a high-quality forage that is widely cultivated in northern China where there is little snow but severe cold in winter. The extreme cold has caused the failure of alfalfa to survive the winter, which is an obstacle to its regeneration and production and leads to serious economic losses. Improving the ability of alfalfa to survive the winter has become an urgent production issue.

The coping responses of alfalfa to freezing conditions in winter can be divided into three distinct phases: acclimation, freezing, and de-acclimation [1, 2]. The initial step of acclimation is growth reduction triggered by the shorter photoperiod and lower temperatures in the fall [3]. Alfalfa accumulates compatible soluble sugars and low-molecular-weight nitrogenous compounds in response to freezing, and there are changes in the cell membrane composition [4, 5] that contribute to improved freezing tolerance [6]. Generally, during acclimation, growing days with temperatures between 0 °C and 5 °C are used as a criterion to estimate the ability of alfalfa to survive [7]. In the subsequent freezing phase, the growth of alfalfa ceases. The crown is the most cold-stress sensitive tissue [8]. When the temperature increases in the following spring, alfalfa loses some of its acquired freezing tolerance through the de-acclimation process [9].

During exposure to low temperatures, plant cells accumulate reactive oxygen species [10], which oxidize the cell membrane and produce malondialdehyde [11]. Consequently, the level of malondialdehyde is an indicator of membrane lipid peroxidation [12]. Under cold stress conditions, the membrane permeability of plant cells increases. In previous studies, the semi-lethal temperature (LT_50_), i.e., the temperature that results in 50% cytochylema leakage, has been used to evaluate plant freezing tolerance and is quantified by measuring electrical conductivity [13]. In response to cold, biochemical changes in plant cells lead to low-temperature acclimation, which can increase the ability of alfalfa to resist adverse environmental conditions. Such biochemical changes include alterations in the contents of soluble carbohydrates and nitrogenous compounds (proteins and amino acids) and the modification of the membrane lipid composition [9, 14, 15]. Metabolomics analyses based on liquid chromatography-mass spectrometry (LC-MS) are effective approaches to identify such changes.

In agricultural production, water-deficit priming is an effective way to increase the freezing tolerance of crops. Similar to the low temperature and short day conditions, water stress can initially trigger a frost-hardening mechanism to induce frost hardiness. This has been demonstrated in red osier dogwood plants (*Cornus stolonifera* Michx.) [16], and even in herbaceous plants such as winter rye (*Secale cereale* L., cv. Puma) [17] and wheat (*Triticum aestivum* L.) [18]. The growth of the legume alfalfa decreases with changes in the photoperiod and declining temperatures in autumn and early winter. Exposing alfalfa to a water deficit increases its winter survival by enhancing its freezing tolerance [19]. Additionally, the effects of a high water level (100% water-holding capacity) and water deficit (50% water-holding capacity) before defoliation on the winter survival of alfalfa have been investigated. The water deficit facilitates root growth and the accumulation of total soluble protein and vegetative storage protein in the taproot, thus increasing the rate of winter survival and spring recovery [20]. In contrast, ample water facilitates vigorous growth and delays the process of cold hardening in early winter [21]. However, there are conflicting opinions on whether a water deficit limits the growth of shoots and roots, as well as the accumulation of nitrogenous compounds in taproots, none of which are conducive to increased cold hardiness [22].

Soil moisture affects the ability of alfalfa to resist freezing. Inspired by cross-adaptation [23], we hypothesized that a continuous water deficit would enhance the freezing tolerance of alfalfa. Therefore, the aims of this study were as follows: 1) to investigate the difference in the freezing tolerance of alfalfa cultivated under two soil-water regimes; and 2) to determine how water deficit affects freezing tolerance using biochemical and metabolomics analyses.

## Methods

### Plant materials

Seeds of alfalfa (cultivar WL353LH, fall dormancy 4.0) were sown in plastic pots (diameter, 15 cm; height, 20 cm). The pots were filled with a mixture of soil, perlite, and vermiculite at a ratio of 100:30:55 (g:g:g). The water-holding capacity of the mixture was 109.7% (g:g). The experiment was conducted in a growth room with the following conditions: 24 ± 2 °C/20 ± 2 °C (light/dark), 60–65% atmospheric relative humidity, and a 12-h light/12-h dark photoperiod with a photosynthetic photon flux density of 350 μmol/m^2^/s. All the pots were watered every day and fertilized once a month with an equal volume of Hoagland’s nutrient solution.

### Experimental treatments

**Fig. 1:**
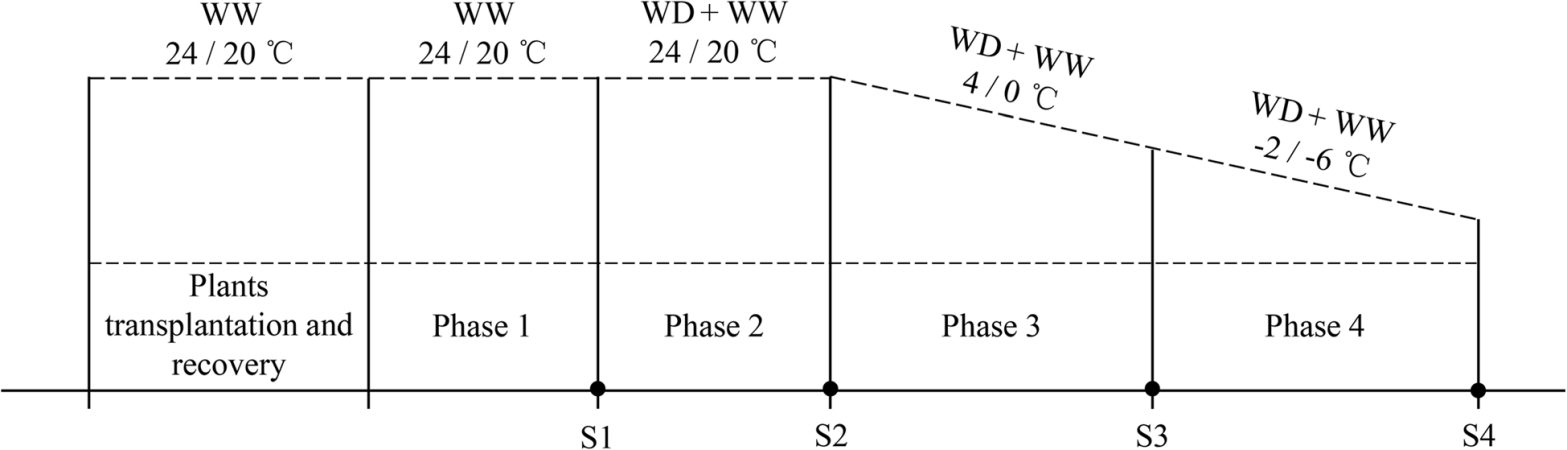
The four experimental phases in this research. The treatments are water-controlled treatments with substrate moisture at 80% (WW) and 25% of water-holding capacity (WD). Incubator temperatures were set for each of the four phases. Black dots S1 to S4 represent sampling time points at the ends of phases

Three months after sprouting, four identical seedlings were selected and transplanted into a polyvinyl chloride pipe (diameter, 10 cm; height, 15 cm). The pipes were filled with the same mixture described in Section 2.1. A total of 42 pipes were prepared for this study, which had two water-regime treatments, each with six biological replicates and four experimental phases. All the pipes were watered to 80% water-holding capacity and then left for 2 weeks in the culture room. Then, the pipes were randomly arranged in an LRH-200-GD (Taihong Medical Instruments, Guangdong, China) low-temperature incubator. The moisture contents of the mixed soil and temperatures in the incubator during the four experiment phases are shown in Fig. 1. The conditions in phase 1 were 80% water-holding capacity and 24/20 °C (light/dark). Plants were grown under these conditions for 1 week, and then samples were collected to represent the original state. In phase 2, the remaining 36 pipes were divided into two groups, the moisture content in the mixed soil of one group was controlled to 25% water-holding capacity (WD) and that of the other group was maintained at 80% water-holding capacity (WW). The temperature in the incubator was set to 24/20 °C (light/dark). Samples were taken on the 3rd d after the WD moisture level was reached. In phase 3, with the soil moisture contents still at WD and WW, the temperature in the incubator was changed from 24/20 °C to 4/0 °C (light/dark), and then samples were taken after 2 days of cold acclimation. In phase 4, the temperature was decreased at a rate of − 2 °C/d, and samples were taken on the next day after the temperature reached − 2/− 6 °C (light/dark). From phase 1 to 4, a 12-h photoperiod with a photosynthetic photon flux density of approximately 150 μmol/m^2^/s was maintained in the incubator.

### Sampling

At each sampling point, as shown in Fig. 1, the above-ground parts were separated from below-ground parts, and then each part was washed with water and surface-dried by blotting with filter paper. We collected crowns within approximately 5-cm below ground as described in previous studies [24–26]. The crown samples were cut into three segments in preparation for the freezing tolerance test, metabolomics assays, and other analyses, and two out of three segments were frozen at − 80 °C until used. After crown sampling, the dry weight of the aboveground parts was determined, and that of the belowground parts was calculated using the total fresh weight and the moisture content of the remaining root tissues.

### Freezing tolerance test

Six replicates of crown samples were prepared for the freezing tolerance test. Crowns were sliced, and the pieces were divided into nine 2-ml centrifuge tubes. The tubes were stored at 4 °C for 2 h and then placed on ice for temporary preservation. The freezing test was carried out in a constant-temperature circulator ZX-5C (Zhixin, Shanghai, China) under nine decreasing temperatures. For the samples collected in phase 1, the freezing temperatures were set to 0, − 2, − 4, − 6, − 8, − 10, − 12, − 14, and − 16 °C. For the samples collected in phase 2, the temperatures were set to − 2, − 4, − 6, − 8, − 10, − 12, − 14, − 16, and − 18 °C. For the samples collected in phases 3 and 4, the temperatures were set to − 4, − 6, − 8, − 10, − 12, − 14, − 16, − 18, and − 20 °C. The samples were subjected to each temperature treatment for 1.5 h in an alcohol bath. One frozen sampling tube was taken out and thawed on ice overnight. The next day, the sampling tubes were thawed at 4 °C for 2 h before the crown slices were transferred to 15-ml tubes and mixed with 5 ml deionized water. The sampling tubes were placed on a gyratory platform shaker HZQ-A (Hengrui Instrument and Equipment, Changzhou, Jiangsu, China) at 120 r/min for 12 h. Electrical conductivity (EL_1_) was measured using a FE38 conductivity meter (Mettler Toledo, Shanghai, China). Crown samples were then autoclaved at 120 °C for 30 min, and electrical conductivity was remeasured (EL_2_). The electrical conductivity of deionized water was determined as EL. The relative electrolyte leakage at a given freezing temperature was calculated as follows:

$$ \mathrm{Relative}\ \mathrm{electrolyte}\ \mathrm{leakage}=\frac{\mathrm{E}{\mathrm{L}}_1-\mathrm{EL}}{\mathrm{E}{\mathrm{L}}_2-\mathrm{EL}}\times 100\%\dots .\dots .\dots (1) $$

The LT_50_ of the crown was calculated as follows:

$$ \mathrm{y}=\frac{\mathrm{A}}{1+\mathrm{B}\times {\mathrm{e}}^{-\mathrm{kx}}}\times 100\%\dots \dots \dots \dots \dots \dots \dots \dots \dots \dots \dots .\dots \dots \dots (2) $$

where y represents the relative electrolyte leakage, x represents the freezing temperature, and A, B, and k are constants.

### Determination of malondialdehyde, carbohydrate, and nitrogenous compound contents

To determine the contents of malondialdehyde, starch, total soluble sugars, total soluble protein, total amino acids, and proline, frozen crown samples (each approximately 0.1 g) were analyzed using the methods described by Draper [27], Yemm [28], Sedmak [29], Rosen [30], and Troll [31] in accordance with the instructions of the appropriate commercial kit (Nanjing Jiancheng Bioengineering Institute, Nanjing, China). The reaction between malondialdehyde and thiobarbituric acid produces a reddish-brown substance having a maximum absorbance value at 532 nm. Thus, the malondialdehyde content was calculated based on the absorbance value. Starch was separated from soluble sugars using 80% ethanol, and then hydrolyzed into glucose using concentrated sulfuric acid. Glucose was then quantified using anthrone colorimetry, and the starch concentration was calculated from the glucose concentration. Soluble sugars were reacted with concentrated sulfuric acid and anthrone to generate blue-green derivatives having maximum absorbance values at 620 nm. The total soluble sugar content was calculated from the absorbance value. The blue compound formed by the reaction between the -NH_3_^+^ of proteins and the Coomassie brilliant blue reagent has a maximum absorbance value at 595 nm. Therefore, the content of total soluble protein was calculated from the absorbance value at 595 nm. The blue-green color compound formed in the reaction of amino acids with Cu^2+^ has a maximum absorbance value at 650 nm; therefore, this was used to calculate total amino acid content. Proline was extracted with sulfosalicylic acid. The product formed in the reaction between proline and acidic ninhydrin solution has a maximum absorbance value at 650 nm; therefore, this value was used to calculate the proline content. After determining the concentrations of these compounds, their values were normalized against the moisture content of root tissues, as described by Sanchez [32]. This normalization of the data allowed us to make meaningful comparisons among groups with different moisture contents.

### Untargeted metabolomics analysis

From phases 2 to 4, six biological replicates were assayed for the metabolomics analysis. The groups were designated as WD_1, 2, and 3, and WW_1, 2, and 3. To evaluate the influence of water deficit on freezing tolerance at normal and low temperatures, comparisons were conducted within phases (WD_1 vs. WW_1, WD_2 vs. WW_2, and WD_3 vs. WW_3) and between phases (WD_2 vs. WD_1, WD_3 vs. WD_2, WW_2 vs. WW_1, and WW_3 vs. WW_2).

#### Metabolite extraction

The frozen crowns were ground to a fine powder in liquid nitrogen. Each sample (60 mg powdered crown tissue) was accurately weighed, and then metabolites were extracted by adding 20 μl internal standard solution (L-2-chlorobenzene alanine prepared with methanol, 0.3 mg/ml) and 0.6 ml of a methanol:water solution (7:3, v:v). Then, the following procedures were conducted: homogenization for 2 min, ultrasonic extraction for 30 min, and incubation for 20 min at − 20 °C. The mixture was centrifuged at a high speed (17,540×g) at 4 °C for 15 min. Finally, 200 μl supernatant was placed in a LC-MS sample vial for testing. To monitor the stability and repeatability of the instrumental analysis, quality control samples were prepared by pooling equal volumes from each sample, and these quality control samples were analyzed along with an equal volume of each sample. The quality control samples were inserted regularly and analyzed after every seven samples.

#### Liquid chromatography-mass spectrometry (LC-MS) analyses

The LC-MS analyses were performed using an ultra-performance liquid chromatography-quadrupole-time of flight mass spectrometry system (Waters Corporation, Milford, MA, USA). For hydrophilic interaction LC separation, the samples were analyzed using an Acquity BEH C18 column (100 mm × 2.1 mm i.d., 1.7 μm; Waters Corporation). Separation was achieved using the following gradient: 5–20% B over 0–2 min, 20–60% B over 2–8 min, 60–100% B over 8–12 min, 100% B over 12–14 min, 100% B–5% B over 14–14.5 min, and holding at 5% B for 1 min, where B is acetonitrile [including 0.1% (v:v) formic acid], A is H_2_O [including 0.1% (v:v) formic acid], and the total proportion of A plus B was 100%. During separation, the flow rate was 0.40 ml/min, the column was maintained at 45 °C, and the injection volume was 3.00 μl. The MS data were collected using a TOF MS equipped with an electrospray ionization source operating in either positive or negative ion mode. The capillary, sampling, and collision voltages were 1.0 kV, 40 V, and 6 eV, respectively. The ion source and solvent removal temperatures were set at 120 °C and 500 °C, respectively, with a desolvation gas flow of 900 l/h. Centroid data were collected from 50 to 1000 *m/z* with a 0.1-s scanning time and a 0.02-s interval.

#### Separation of metabolites and bioinformatics analyses

The raw mass spectrum data were converted using the processing software Progenesis QI (Waters Corporation) for baseline filtration, peak identification, integration, retention time correction, peak alignment, and uniformization. Next, following the method described by Sanchez [32], all the peak intensity values were normalized against the moisture contents of root tissues to allow for comparisons among different groups. Finally, we obtained a data matrix (.xls) that contained the retention time, mass charge ratio, and peak intensity. The data matrix was converted using SIMCA-P software (V14, Umetrics, Umea, Sweden) to perform principal component, partial least squares discriminant, and orthogonal partial least squares discriminant analyses. Differential metabolites were determined based on the combination of a statistically significant threshold of variable influence on projection (VIP) value obtained from the orthogonal partial least squares discriminant analysis and Student’s t-test (*P*-value) of the raw data. The metabolites with VIP > 1.5 and *P* < 0.05 were considered statistically significant. A qualitative analysis of each metabolite and the acquisition of its compound identification number (CID) was performed using the Human Metabolome Database (http://www.hmdb.ca/), Lipid Maps (http://www.lipidmaps.org/), and Metabolites Biological Role (http://csbg.cnb.csic.cs/mbrole). Then, a pathway annotation analysis was conducted using the CID in the Kyoto Encyclopedia of Genes and Genomes (KEGG, https://www.genome.jp/kegg/pathway.html) database. Metabolite classification, prominent metabolic pathway detection, and enrichment analyses were also performed.

### Data analyses

The biomass, malondialdehyde, carbohydrate, nitrogenous compound, and LT_50_ data were subjected to analyses of variance between phases and within phases using SPSS 20.0 (SPSS Inc., Chicago, IL, USA). Multiple range tests were performed using least significant differences, and differences were considered significant at *P* < 0.05.

## Results

### Dry weights of aboveground and belowground parts

**Fig. 2:**
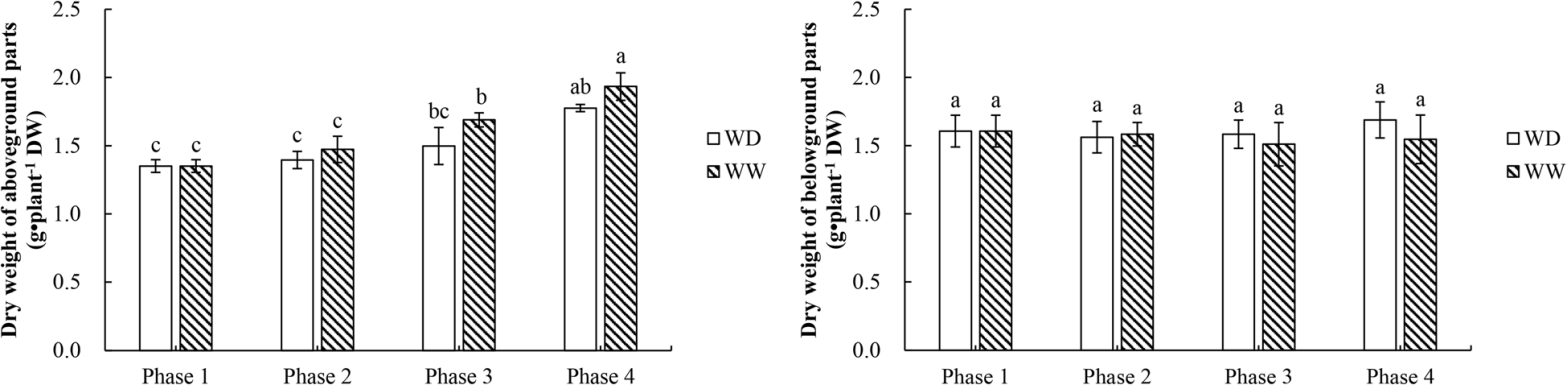
Dry weights of aboveground and belowground alfalfa parts in the four experimental phases. WW and WD: substrate moisture at 80 and 25% of water-holding capacity, respectively. The significances of differences between and within phases were evaluated using a multiple-range test. Different letters indicate significant differences (*P* < 0.05)

To evaluate the effects of alfalfa growth on its freezing tolerance, the dry weights of aboveground and belowground parts were determined (Fig. 2). The dry weight of the aboveground parts increased significantly from phase 1 to 4, but no significant difference was detected between the two water-controlled treatments. The dry weight of the belowground parts only changed slightly from phase 1 to 4, and no remarkable difference was detected between the two water-controlled treatments.

### Semi-lethal temperatures

**Fig. 3:**
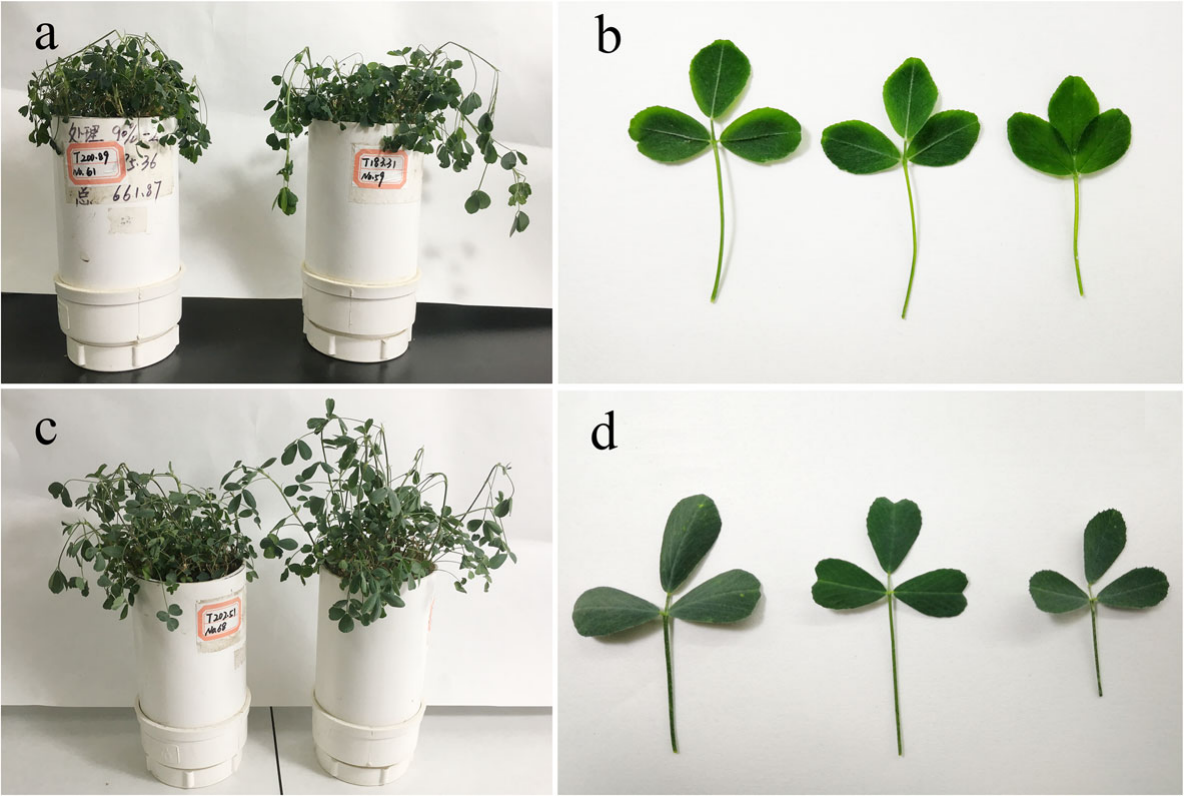
Phenotypes of alfalfa after freezing in phase 4. WW and WD: substrate moisture at 80 and 25% of water-holding capacity, respectively. Phenotypes of alfalfa in WW (**a** and **b**) and in WD (**c** and **d**)

**Fig. 4:**
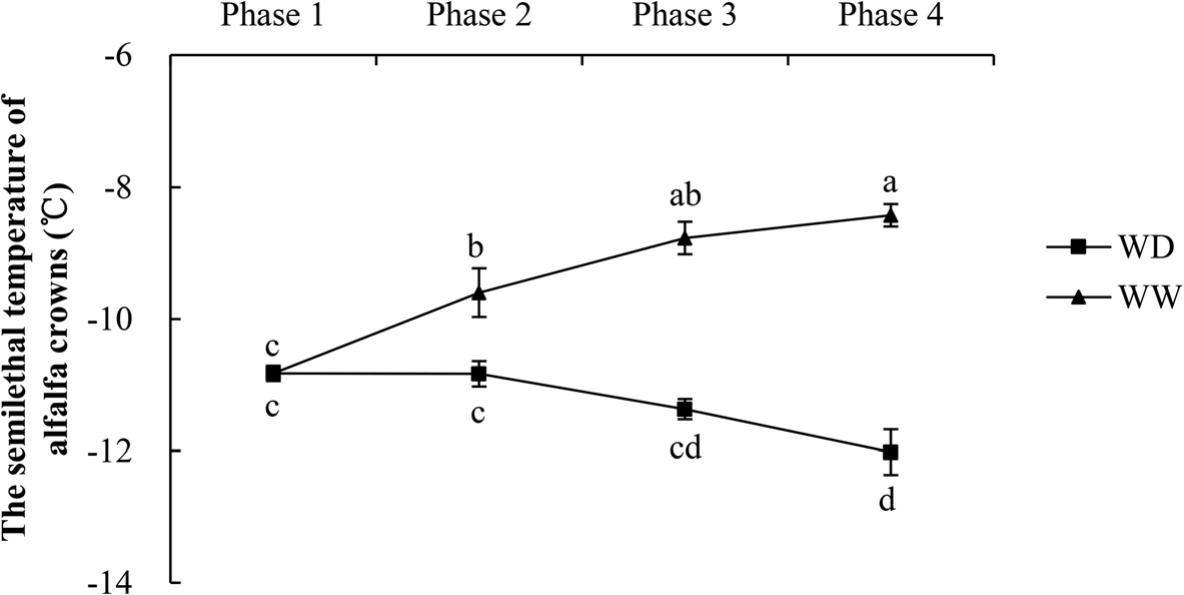
Semi-lethal temperature, LT_50_, of alfalfa crowns collected in WD and WW. WW and WD: substrate moisture at 80 and 25% of water-holding capacity, respectively. The significances of differences between and within phases were evaluated using a multiple-range test. Different letters indicate significant differences (*P* < 0.05)

Judging from the phenotypes at the end of phase 4 (Fig. 3a–d), alfalfa plants in WW were damaged more seriously than those in WD, as demonstrated by the higher relative electrolyte leakage of their leaves (75% vs. 33%). The WD treatment significantly improved the ability of alfalfa to resist low temperatures. As shown in Fig. 4**,** the LT_50_ of crowns increased gradually from phase 1 to 4 in WW, but not in WD. At the end of phase 2, the LT_50_ was significantly lower in WD than in WW, and the difference increased after exposure to 4/0 °C and − 2/− 6 °C. From phase 1 to 4, the LT_50_ of the crowns increased by 2.40 °C (or 22.2%) in WW, but decreased by 1.19 °C (or 11.0%) in WD. At normal temperatures, water deficit increased the freezing tolerance of alfalfa, and freezing tolerance was further enhanced by exposure to lower temperatures.

### Malondialdehyde contents

**Fig. 5:**
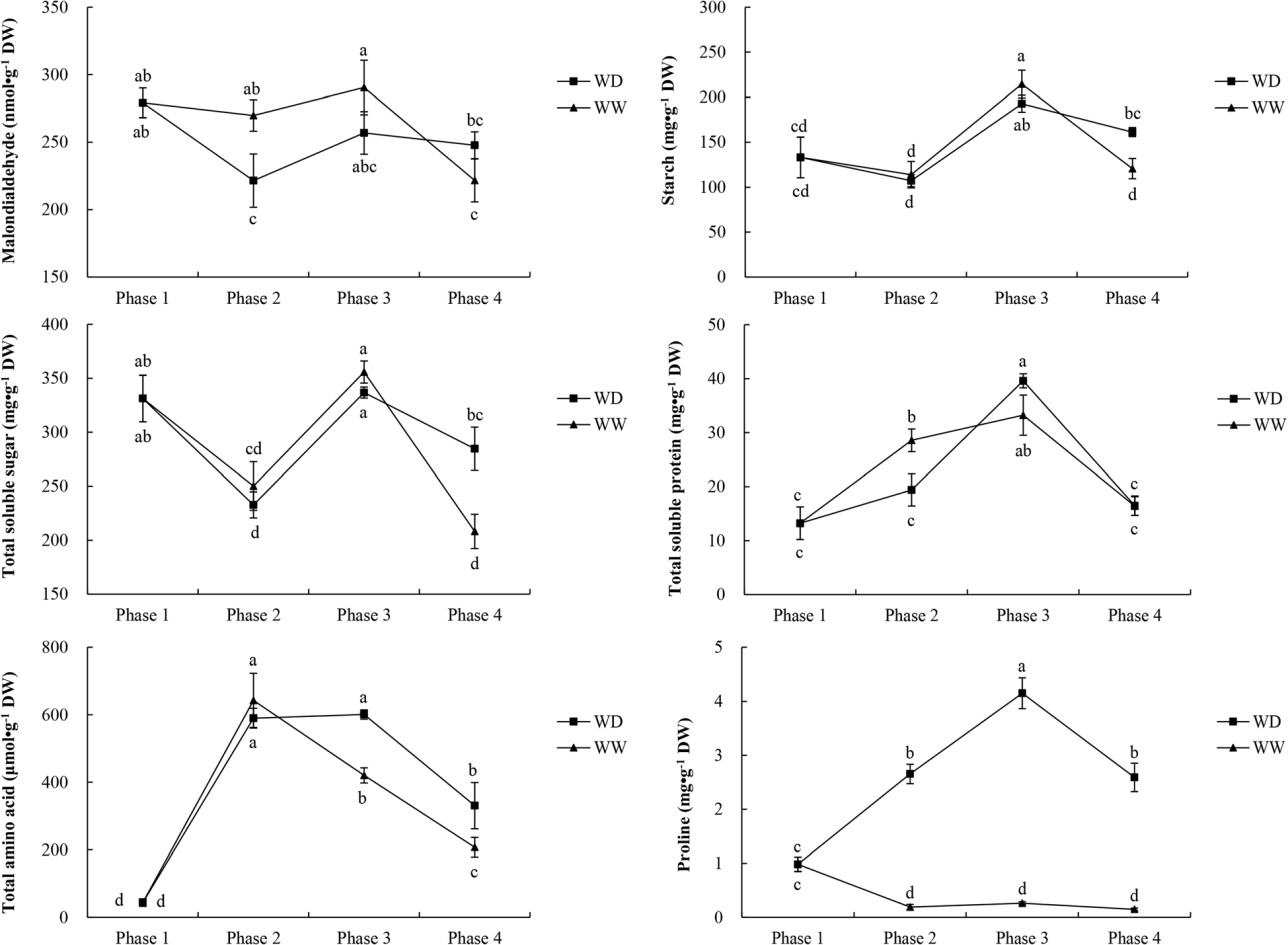
Changes in the contents of malondialdehyde, carbohydrates (starch and total soluble sugar), and nitrogenous compounds (total soluble protein, total amino acids, and proline) in the four phases. WW and WD: substrate moisture at 80 and 25% of water-holding capacity, respectively. The significances of differences between and within phases were evaluated using a multiple-range test. Different letters indicate significant differences (*P* < 0.05)

The concentrations of malondialdehyde in alfalfa crowns in the two treatments are shown in Fig. 5. At the end of phase 2, the malondialdehyde content in WW was significantly higher than that in WD. However, at the end of phases 3 and 4, the malondialdehyde contents did not differ significantly between the two water-controlled treatments.

### Carbohydrates and nitrogenous compounds

The concentrations of five compounds, including carbohydrates and nitrogenous compounds, in plants during the WD and WW treatments are shown in Fig. 5. The contents of starch and total soluble sugar increased in phase 3 and decreased in phase 4. At the end of phase 4, the contents of starch and total soluble sugar were significantly higher in WD than in WW. The total soluble protein concentration at the end of phase 2 was significantly higher in WW than in WD. Subsequently, the soluble protein content in WD significantly increased in phase 3 and decreased in phase 4. There were no significant differences in soluble protein contents between the two treatments at the ends of phases 3 and 4. The total amino acid content was higher in WD than in WW, with significantly higher levels in WD than in WW at the ends of phases 3 and 4. Compared with the WW treatment, the WD treatment led to a dramatic increase in the proline content in phase 2. The proline content in WD increased during cold acclimation (phase 3) and then decreased under freezing conditions (phase 4). On the whole, at the end of phases 2, 3, and 4, the proline contents were significantly (*P* < 0.05) higher in WD than in WW.

### Differential metabolites

#### Separation of differential metabolites

**Fig. 6:**
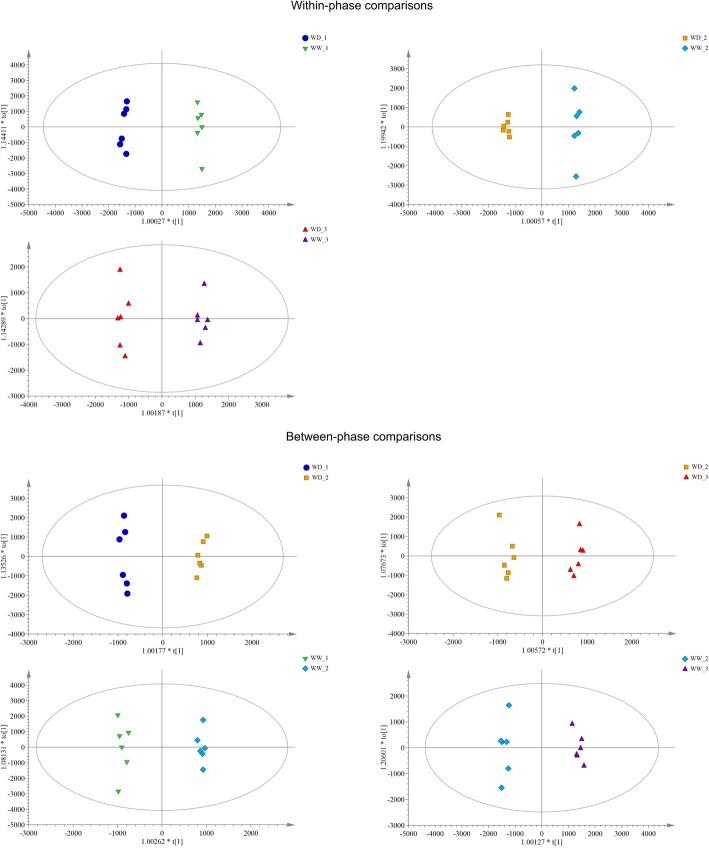
Scatter plots of scores of the orthogonal partial least squares discriminant analysis for identified differential metabolites in the seven comparisons. WW and WD: substrate moisture at 80 and 25% of water-holding capacity, respectively. WD_1, 2, 3 and WW_1, 2, 3 represent samples collected at the ends of phases 2, 3, and 4, respectively

As shown in Fig. 6, the significant differential metabolites between WD and WW were well separated based on the criteria VIP > 1.5 and *P* < 0.05. Table 1 and Additional file 1 shows the number and detail information of differential metabolites, reapectively. In within-phase comparisons, three upregulated differential metabolites were found in WD_1 vs. WW_1, and 25 were found in WD_3 vs. WW_3. In between-phase comparisons, there were more upregulated differential metabolites in WD_2 vs. WD_1 than in WD_3 vs. WD_2, while there were similar numbers in WW_2 vs. WW_1 and WW_3 vs. WW_2. Detailed information on the upregulated differential metabolites in the seven comparison groups, including classification and fold changes, are summarized in Additional file 2.

**Table 1:** Numbers of differential metabolites. All = Upregulated+Downregulated. Only some metabolites had CIDs required for further annotation in KEGG

	Within-phase comparisons			Between-phase comparisons			
	WD_1 vs WW_1	WD_2 vs WW_2	WD_3 vs WW_3	WD_2 vs WD_1	WD_3 vs WD_2	WW_2 vs WW_1	WW_3 vs WW_2
All	131	112	115	85	39	46	174
Upregulated	3	0	25	74	4	23	19
Downregulated	128	112	90	11	35	23	155
CID	19	18	17	12	5	10	34
Annotated in KEGG	19	18	17	12	5	10	34

#### Classification of differential metabolites

**Fig. 7:**
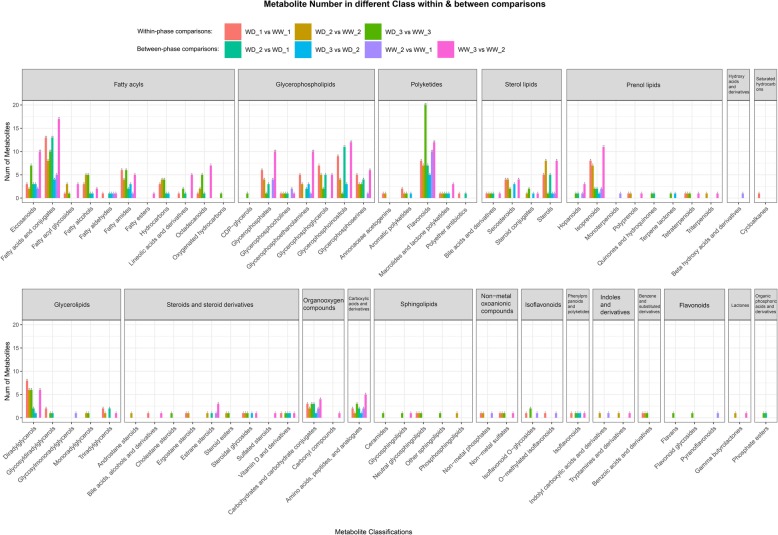
Numbers of differential metabolites at the class and subclass levels. Names on the abscissa represent subclasses, gray parts above the plot represent classes. Within-phase and between-phase comparisons revealed metabolites affected by water deficit and low temperature, respectively. WW and WD: substrate moisture at 80 and 25% of water-holding capacity, respectively. WD_1, 2, 3 and WW_1, 2, 3 represent samples collected at the ends of phases 2, 3, and 4, respectively

As shown in Fig. 7, the differential metabolites in seven comparisons were categorized into 20 classes and 72 subclasses. Judging from the proportion of differential metabolites in each class out of the total differential metabolites (Additional file 3), fatty acyls, glycerolipids, glycerophospholipids, polyketides, prenol lipids, steroids and steroid derivatives, sterol lipids, carboxylic acids and derivatives, and organooxygen compounds were the most active metabolites. At the superclass level, the active metabolites were classified as lipid and lipid-like molecules, as well as carboxylic acids and their derivatives, and organooxygen compounds. At the subclass level, there were approximately 18 species of active differential metabolites, including fatty acids and their conjugates, flavonoids, eicosanoids, glycerophosphoinositols, isoprenoids, and carbohydrates and carbohydrate conjugates. In summary, the differential metabolites identified in the seven comparisons mainly included carbohydrates, amino acids, fatty acids, glycerophospholipids, and flavonoids.

In the three within-phase comparisons, the upregulated differential metabolites included lipid and lipid-like molecules and organic acids and their derivatives at the superclass level (Additional file 2). At the subclass level, these metabolites could be classified into eicosanoids, fatty acids and their conjugates, linoleic acid and its derivatives, octadecanoids, glycerophosphoserines, amino acids, and peptides and analogues. In this study, all of these metabolites were upregulated under water-deficit conditions.

In the four between-phase comparisons, 34 types of upregulated differential metabolites were identified at the subclass level. In addition to those mentioned in the within-phase comparisons, the other upregulated metabolites included glycerophosphates, glycerophosphocholines, glycerophosphoethanolamines, glycerophosphoglycerols, glycerophosphoinositols, flavonoids, and carbohydrates and carbohydrate conjugates. These upregulated differential metabolites were lipid and lipid-like molecules (at the superclass level), amino acids, peptides and analogues, and carbohydrates and carbohydrate conjugates (at the subclass level). All of these metabolites showed increased abundance levels at declining temperatures.

#### KEGG analysis

**Fig. 8:**
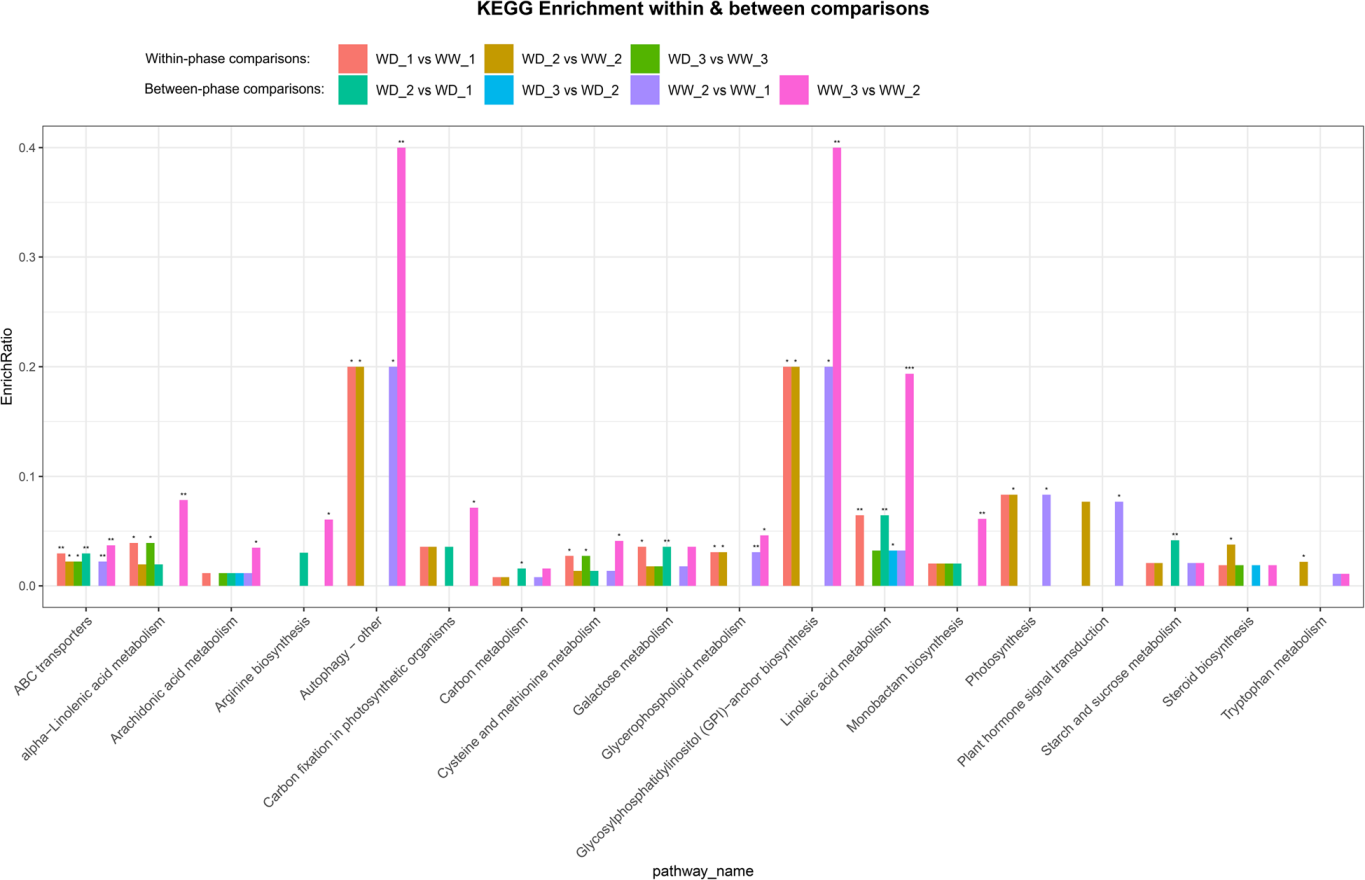
Enrichment ratios of 18 significantly enriched pathways. Asterisks represent significance at *P* < 0.05 (*), *P* < 0.01 (**), and *P* < 0.001 (***). Within-phase and between-phase comparisons revealed pathways affected by water deficit and low temperatures, respectively. WW and WD: substrate moisture at 80 and 25% of water-holding capacity, respectively. WD_1, 2, 3 and WW_1, 2, 3 represent samples collected at the ends of phases 2, 3, and 4, respectively

Some of the separated differential metabolites had CID numbers, which allowed them to be annotated in the KEGG database. A total of 18 prominent pathways were significantly enriched (*P* < 0.05, *P* < 0.01, and *P* < 0.001) with identified differential compounds (Fig. 8). These pathways were mainly involved in the biosynthesis and metabolism of carbohydrates, unsaturated fatty acids, amino acids, and glycerophospholipids. The other pathways that were not significantly enriched, as determined by the KEGG analysis, are listed in Additional file 4.

#### Differential metabolites involved in prominent pathways

At the class level, the differential metabolites involved in 18 prominent pathways included nonmetal oxoanionic compounds, glycerophospholipids, fatty acyls, organooxygen compounds, steroids and steroid derivatives, carboxylic acids and derivatives, and indoles and their derivatives (Table 2). At the superclass level, these metabolites included lipid and lipid-like molecules, organic acids and derivatives, and organic oxygen compounds. At the subclass level, they were mainly glycerophosphocholines, glycerophosphoethanolamines, linoleic acid and its derivatives, eicosanoids, fatty acids and conjugates, amino acids, peptides and analogues, and carbohydrates and carbohydrate conjugates.

**Table 2:** Fold changes of the metabolites involved in 18 pathways identified as being significantly enriched in the KEGG analysis. “/” indicates that the metabolite or a significant difference was not detected

Metabolites	Subclass	Within-phase comparisons			Between-phase comparisons			
		WD_1 vs WW_1	WD_2 vs WW_2	WD_3 vs WW_3	WD_2 vs WD_1	WD_3 vs WD_2	WW_2 vs WW_1	WW_3 vs WW_2
*Nonmetal oxoanionic compounds*								
Phosphoric acid	Nonmetal phosphates	0.4766	0.4204	/	/	/	0.7124	/
Sulfate	Nonmetal sulfates	0.6713	0.6650	0.7656	/	/	/	0.8236
*Fatty acyls*								
13-oxo-octadecadienoic acid	Linoleic acids and derivatives	/	/	/	/	/	/	0.4233
13S-hydroxy-octadecadienoic acid	Linoleic acids and derivatives	/	/	/	/	/	/	0.4576
20-hydroxy-leukotriene B4	Eicosanoids	/	/	/	/	/	/	0.4847
9,10-dihydroxy-12-octadec-enoic acid	Fatty acids and conjugates	1.4183	/	/	0.7178	/	/	0.5667
9,12,13-trihydroxy-octadec-10-enoic acid	Fatty acids and conjugates	1.4510	/	2.0848	/	/	1.5159	0.3778
9S,11R,15S-trihydroxy-2,3-dinor-13E-prostaenoic acid-cyclo[8S,12R]	Eicosanoids	/	/	1.6657	/	/	/	0.4795
Alpha-linolenic acid	Linoleic acids and derivatives	/	/	/	/	/	/	0.4510
Arachidonic acid (d8)	Fatty acids and conjugates	/	/	/	1.9797	0.5671	/	0.2218
Norlinolenic acid	Fatty acids and conjugates	0.5674	/	1.8503	1.7476	/	/	0.3516
Traumatic acid	Fatty acids and conjugates	0.5335	0.5854	/	/	/	/	0.6102
Traumatin	Fatty acids and conjugates	/	/	3.3487	/	/	/	0.1040
*Glycerophospholipids*								
Lysophosphatidylcholine(16:0)	Glycerophosphocholines	/	/	/	/	/	2.1402	/
Phosphatidyl alcohol(16:0/18:2(9Z,12Z))	Glycerophosphates	0.7050	0.6145	/	/	/	/	0.6955
Phosphatidyl ethanolamine(16:1(9Z)/P-18:1(11Z))	Glycerophosphoethanolamines	0.6389	0.6606	/	/	/	/	0.6178
Phosphatidyl ethanolamine(18:3(6Z,9Z,12Z)/P-18:0)	Glycerophosphoethanolamines	/	/	/	/	/	1.3388	0.6226
*Steroids and steroid derivatives*								
Campesterol	Ergostane steroids	0.5078	0.5925	/	/	/	/	/
Cholesterol ester(20:1(11Z))	Steroid esters	/	0.4889	0.4198	/	/	/	/
*Carboxylic acids and derivatives*								
L-arginine	Amino acids, peptides, and analogues	/	/	/	/	/	/	0.1530
L-aspartic acid	Amino acids, peptides, and analogues	/	/	/	2.4269	/	/	0.2733
L-cystathionine	Amino acids, peptides, and analogues	0.4052	/	0.2932	/	/	0.4034	3.2124
*Organooxygen compounds*								
D-glucose	Carbohydrates and carbohydrate conjugates	/	/	/	1.3310	/	1.2303	/
D-maltose	Carbohydrates and carbohydrate conjugates	/	/	/	1.1766	/	/	/
Fructose 6-phosphate	Carbohydrates and carbohydrate conjugates	0.5245	0.4511	/	/	/	/	0.6804
Maltotriose	Carbohydrates and carbohydrate conjugates	0.4487	0.7361	0.5299	/	/	0.5619	1.7477
Raffinose	Carbohydrates and carbohydrate conjugates	0.4885	/	0.7346	1.6008	/	/	1.2806
*Indoles and derivatives*								
Indoleacetic acid	Indolyl carboxylic acids and derivatives	/	0.6223	/	/	/	1.5008	/
5-methoxytryptamine	Tryptamines and derivatives	/	0.6575	/	/	/	/	0.4630
*Others*								
Alpha-dimorphecolic	Others	/	/	/	/	/	/	0.3647

## Discussion

### Effects of water deficit on freezing tolerance and membrane permeability

A previous study showed that, either at 20/15 °C (day/night) or at cold/freezing temperatures, alfalfa growing at a 25% field-water capacity has a higher freezing tolerance than alfalfa growing at 60 and 100% field-water capacities, which suggested that a moderate water deficit could enhance freezing tolerance [19]. In this study, the LT_50_ of alfalfa was significantly lower in the WD treatment than in the WW treatment. An analysis of the changes in aboveground and belowground dry weights confirmed that the differences in LT_50_ between WD and WW were not caused by differences in alfalfa growth (Fig. 2). Thus, our results also support the conclusion that water deficit significantly enhances the freezing tolerance of alfalfa at normal temperatures and that this effect is strengthened after exposure to lower temperatures.

The level of malondialdehyde reflects the degree of membrane lipid peroxidation in the cell membrane [12]. The malondialdehyde content differed significantly between WW and WD at the end of phase 2, but not and the ends of phases 3 and 4. At the ends of phases 2 and 3, the contents of malondialdehyde were slightly higher in WW than in WD. Greater damage to the cellular membrane caused by lipid peroxidation was evidenced by the higher membrane permeability and cytochylema leakage in WW than in WD (Fig. 4).

### Correlations between biochemical changes and freezing tolerance

Freezing stress inhibits plant cellular metabolism and reduces water uptake, thus inducing dehydration or osmotic stress [33, 34]. Previous studies have reported that biochemical substances, including those related to osmotic regulation, accumulate in the crown and taproot when alfalfa is exposed to low temperatures [35–37]. In addition, a previous study reported that the soluble protein content in alfalfa is affected by low temperature, cold acclimation, or freezing [15]. In this study, although the soluble protein content changed significantly during the processes of cold acclimation and freezing (phases 3 and 4), it did not differ significantly between the two treatments. This indicates that soluble protein had a measurable response to cold and freezing, but not to water deficit. Soluble sugars can act as cryoprotectants by preventing sudden osmotic shock and ice formation, and they help to stabilize the integrity of macromolecules and the plasma membrane during freezing-induced desiccation [38–42]. In this study, the total soluble sugar content differed significantly between the WW and WD treatments. The contents of starch and total soluble sugar decreased in freezing phase 4, but some soluble sugars may have remained trapped in the insoluble starch and would, therefore, be unavailable to contribute to the osmotic potential of the cell. Alfalfa accumulates amino acids, such as proline, which act as osmotic regulatory molecules in response to drought stress [43, 44]. Although the role of proline in the acquisition of freezing tolerance remains unclear, evidence from transgenic experiments supports the adaptive value of stress-induced proline [24].

Exposing alfalfa to low, non-freezing temperatures (about 0–5 °C) induces morphological, physiological, and biochemical changes that improve cold hardiness and lead to the acquisition of freezing tolerance [45, 46]. In this study, plants were subjected to 2 d of a cold-acclimation treatment at 4/0 °C (light/dark). Although the contents of total soluble sugar and total soluble protein increased during this period, alfalfa plants in the WW treatment showed no significant increase in freezing tolerance. This may be because of inadequate acclimation. That is, the short period of acclimation (2 d) at 4/0 °C (light/dark) after reducing the temperatures from 24/20 °C to 4/0 °C (light/dark) may have been inadequate. Nevertheless, the freezing tolerance of alfalfa in the WD treatment increased significantly during this cold-acclimation treatment (phase 3), possibly because of the significant increase in the proline content. During acclimation, the contents of total soluble sugar and total soluble protein increased in both the WW and WD treatments. In addition, at the end of phase 4, the soluble sugar content differed significantly between the WW and WD treatments. Thus, soluble sugars may also play roles in improving the freezing tolerance of alfalfa. After exposure to low temperatures in phase 4, alfalfa’s freezing tolerance decreased in WW but increased in WD, providing more evidence that water deficit improves the freezing tolerance of alfalfa.

In summary, the contents of soluble sugars and amino acids, including proline, increased under water-deficit and low-temperature conditions. These substances may contribute to the enhancement of freezing tolerance under moderate water-deficit conditions.

### Contributions of differential metabolites to freezing tolerance

Differential metabolites were classified to evaluate their contributions to the difference in freezing tolerance between plants receiving the WD and WW treatments. Within-phase comparisons were performed to detect correlations between differential metabolites and the freezing tolerance of alfalfa under the two water regimes. The biosynthesis and metabolism of lipid and lipid-like molecules and amino acids, peptides, and analogues were greatly enhanced, and the abundance levels of these compounds increased under water-deficit conditions. Plasma membranes are the sites of temperature sensing and major injury upon exposure to low temperature [7, 47]. Low temperatures change the membrane lipid phase from highly fluid to a rigid gel in which lipids are closely packed and highly ordered. The rigid gel phase can render the membrane more permeable and prone to rupture [48]. However, for plants to survive cold or chilling, they must be able to maintain the structural and functional integrity of the cellular membranes. Changes in lipid composition are associated with changes in the cryostability of the plasma membrane [47, 49]. Glycerophosphoserines, a class of glycerophospholipids, were upregulated under water-deficit conditions, as determined by the within-phase comparisons. Glycerophospholipids consist of phosphate and two fatty acids or fatty alcohols [50] and are the dominant component of biological membranes in plants [51]. Within-phase comparisons identified that two unsaturated fatty acids, eicosanoid and linoleic, were upregulated under water-deficit conditions. Unsaturated fatty acids may help maintain the fluidity of membranes owing to the presence of *cis* double bonds. An increase in the proportion of unsaturated fatty acids enhances the integrity/stability and cellular functions of membranes during cold or freezing stress [47, 52]. Furthermore, very low temperatures resulting in freezing injury also target the plasma membranes, and higher proportions of fatty acids in the membrane lipids can enhance membrane cryotolerance and stability [53]. Proline betaine, an important osmoprotectant and the main betaine identified in alfalfa [54], was also upregulated during the water-deficit treatment. Thus, both membrane stabilization as a result of changes in lipid composition and increased osmotic regulation might play important roles in enhancing the freezing tolerance of alfalfa under water-deficit conditions.

Understanding the relationships between metabolites and freezing tolerance following cold acclimation and freezing temperatures can shed light on the mechanisms by which water deficit enhances freezing tolerance. In the four between-phase comparisons, the upregulated metabolites were mainly lipid-and lipid-like molecules (at the superclass level), and amino acids, peptides and analogues, and carbohydrates and carbohydrate conjugates (at the subclass level). In addition to the metabolites discussed in the within-phase comparisons, carbohydrates (including D-glucose, raffinose, maltotetraose, maltotriose, and D-maltose) and flavonoids showed increases in abundance levels at declining temperatures. Soluble sugars play an important role in enhancing freezing tolerance because of their cryoprotective function [38, 39]. A variety of functions have been reported for flavonoids. For example, they function as nonenzymatic antioxidants [55] to alleviate oxidative stress caused by salinity [56], ultraviolet light [56], and heat [57]. Transcriptomic modifications indicative of enhanced flavonoid biosynthesis induced by cold stress have also been reported for *Citrus sinensis* L. [58]. Additionally, the flavonoids that accumulated during cold acclimation play functional roles in enhancing the freezing tolerance of *Arabidopsis thaliana* [59]. In our study, we detected differential metabolites that were upregulated during cold acclimation and freezing. Our results suggest that these metabolites play important roles in enhancing the freezing tolerance of alfalfa.

## Conclusion

In our study, a continuous water deficit treatment significantly enhanced the freezing tolerance of alfalfa compared with a well-watered treatment. Increases in the contents of total soluble sugars and total amino acids, including proline, may have contributed to the difference in LT_50_ between plants receiving the two treatments. The upregulation of lipids and lipid-like molecules (e.g., fatty acids, unsaturated fatty acids, and glycerophospholipids) and amino acids (e.g., proline betaine), as well as the enhancement of related metabolic pathways, may also have contributed to the enhanced freezing tolerance of alfalfa under water-deficit conditions. Other compounds that showed increased abundance levels at low temperatures included flavonoids and carbohydrates, such as D-maltose, D-glucose, raffinose, maltotetraose, and maltotriose. These results indicate that water management can improve the freezing tolerance of alfalfa and is an effective strategy to increase the winter survival of alfalfa crops in northern China.

## Supplementary information

**Additional file 1.** Detailed information for all the differential metabolites detected in the seven comparisons.

**Additional file 2.** Fold changes in differential metabolites upregulated in seven comparison groups.

**Additional file 3.** Proportions of differential metabolites in each class/subclass level out of all the differential metabolites in each comparison.

**Additional file 4.** All the pathways identified in the KEGG enrichment analysis.

**Additional file 5.** The raw data for dry weight, moisture content of roots, LT_50_, malondialdehyde, starch, total soluble sugar, total soluble protein, total amino acid and proline in the two treatments at the ends of the four phases.

**Additional file 6.** The raw data for all the metabolites detected in the seven comparisons.

## Data Availability

All the data generated or analyzed during this study are included in this published article (Additional file 1, Additional file 2, Additional file 3, Additional file 4, Additional file 5, Additional file 6).

## Abbreviations

CID: Compound identification number
KEGG: Kyoto Encyclopedia of Genes and Genomes
LC-MS: Liquid chromatography-mass spectrometry
LT_50_: Semilethal temperature resulting in 50% cytochylema leakage
VIP: Variable influence on projection value
WD: Water-deficit treatment in which the growth moisture was maintained at 25% water-holding capacity
WW: Well-watered treatment in which the growth moisture was maintained at 80% water-holding capacity

## References

[1] Li W, Wand R, Li M, Li L, Wang C, Welti R, Wang X (2008). Differential degradation of extraplastidic and plastidic lipids during freezing and post-freezing recovery in Arabidopsis thaliana. J Biol Chem.

[2] Trischuk RG, Schilling BS, Low NH, Gray GR, Gusta LV (2014). Cold acclimation, de-acclimation and re-acclimation of spring canola, winter canola and winter wheat: the role of carbohydrates, cold-induced stress proteins and vernalization. Environ Exp Bot.

[3] Stout DG, Hall WJ (1989). Fall growth and winter survival of alfalfa in interior British Columbia. Can J Plant Sci.

[4] Korn M, Gärtner T, Erban A, Kopka J, Selbig J, Hincha DK (2010). Predicting Arabidopsis freezing tolerance and heterosis in freezing tolerance from metabolite composition. Mol Plant.

[5] Winfield MO, Lu CG, Wilson ID, Coghill JA, Edwards KJ (2010). Plant responses to cold: Transcriptome analysis of wheat. Plant Biotechnol J.

[6] Chen J, Han G, Shang C, Li J, Zhang H, Liu F, Wang J, Liu H, Zhang Y (2015). Proteomic analyses reveal differences in cold acclimation mechanisms in freezing-tolerant and freezing-sensitive cultivars of alfalfa. Front Plant Sci.

[7] Thomashow MF (1999). Plant cold acclimation: freezing tolerance genes and regulatory mechanisms. Annu Rev Plant Physiol Plant Mol Biol.

[8] Pembleton KC, Volenec JJ, Rawnsley RP, Donaghy DJ (2010). Partitioning of taproot constituents and crown bud development are affected by water deficit in regrowing alfalfa (*Medicago sativa* L.). Crop Sci.

[9] Guy CL (1990). Cold acclimation and freezing stress tolerance: role of protein metabolism. Annu Rev Plant Physiol Plant Mol Biol.

[10] Janská A, Marˇsík P, Zelenková S, Ovesná J (2010). Cold stress and acclimation what is important for metabolic adjustment?. Plant Biol.

[11] Uemura M, Steponkus PL (1999). Cold acclimation in plants: relationship between the lipid composition and the cryostability of the plasma membrane. J Plant Res.

[12] Hu Y, Cao J, Liu P, Guo D, Wang Y, Yin J, Zhu Y, Rahman K (2011). Protective role of tea polyphenols in combination against radiation-induced haematopoietic and biochemical alterations in mice. Phytother Res.

[13] Yang Y, Jia Z, Chen F, Sang Z, Ma L (2015). Comparative analysis of natural cold acclimation and deacclimation of two Magnolia species with different winter hardiness. Acta Physiol Plant.

[14] Theocharis A, Clément C, Barka EA (2012). Physiological and molecular changes in plants grown at low temperatures. Planta..

[15] Dhont C, Castonguay Y, Nadeau P, Belanger G, Drapeau R, Laberge S, Avice JC, Chalifour FP (2006). Nitrogen reserves, spring regrowth and winter survival of field-grown alfalfa (Medicago sativa) defoliated in the autumn. Ann Bot.

[16] Chen H, Li PH (1978). Interactions of low temperature, water stress, and short days in the induction of stem frost hardiness in red osier dogwood. Plant Physiol.

[17] Siminovitch D, Cloutier Y (1982). Twenty-four-hour induction of freezing and drought tolerance in plumules of winter rye seedlings by desiccation stress at room temperature in the dark. Plant Physiol.

[18] Cloutier Y, Siminovitch D (1982). Correlation between cold- and drought-induced frost hardiness in winter wheat and rye varieties. Plant Physiol.

[19] Paquin R, Mehuys GR (1980). Influence of soil moisture on cold tolerance of alfalfa. Can J Plant Sci.

[20] Gorka E, Juan IJ, Manuel SD, Avice JC, Ourry A (2007). Effect of drought, elevated CO_2_ and temperature on accumulation of N and vegetative storage proteins (VSP) in taproot of nodulated alfalfa before and after cutting. Plant Sci.

[21] McKenzie JS, McLean GE (1980). Changes in the cold hardiness of alfalfa during five consecutive winters at Beaverlodge, Alberta. Can J Plant Sci.

[22] Justes E (2002). Thie’beau P, Avice JC, Lemaire G, Lemaire G, Volenec JJ, Ourry a. influence of summer sowing dates, N fertilization and irrigation on autumn VSP accumulation and dynamics of spring regrowth in alfalfa (Medicago sativa L.). J Exp Bot.

[23] Hossain MA, Li Z, Hoque TS, Burritt DJ, Fujita M, Munné-Bosch S (2018). Heat or cold priming-induced cross-tolerance to abiotic stresses in plants: key regulators and possible mechanisms. Protoplasma..

[24] Castonguay Y, Bertrand A, Michaud R, Laberge S (2011). Cold-induced biochemical and molecular changes in alfalfa populations selectively improved for freezing tolerance. Crop Sci.

[25] Bertrand A, Bipfubusa M, Castonguay Y, Rocher S, Szopinska-Morawska A, Papadopoulos Y, Renaut J (2016). A proteome analysis of freezing tolerance in red clover (*Trifolium pratense* L.). BMC Plant Biol.

[26] Liu Z, Yang G, Li X, Yan Y, Sun J, Gao R, Sun Q, Wang Z (2016). Autumn dormancy regulates the expression of cas18, vsp and corF genes during cold acclimation of lucerne (Medicago sativa L.). Crop Pasture Sci.

[27] Draper HH, Hadley M (1990). Malondialdehyde determination as index of lipid peroxidation. Methods Enzymol.

[28] Yemm EW, Willis AJ (1954). The estimation of carbohydrates in plant extracts by anthrone. Biochem J.

[29] Sedmak JJ, Grossberg SE (1977). A rapid, sensitive, and versatile assay for protein using Coomassie brilliant blue G250. Anal Biochem.

[30] Rosen H (1957). A modified ninhydrin colorimetric analysis for amino acids. Arch Biochem Biophys.

[31] Troll W, Lindsley J (1955). A photometric method for the determination of proline. J Biol Chem.

[32] Sanchez DH, Schwabe F, Erban A, Udvardi MK, Kopka J (2012). Comparative metabolomics of drought acclimation in model and forage legumes. Plant Cell Environ.

[33] Chinnusamy V, Zhu J, Zhu J (2007). Cold stress regulation of gene expression in plants. Trends Plant Sci.

[34] Arias NS, Bucci SJ, Scholz FG, Goldstein G (2015). Freezing avoidance by supercooling in Olea europaea cultivars: the role of apoplastic water, solute content and cell wall rigidity. Plant Cell Environ.

[35] Tarkowski ŁP, Van den Ende W (2015). Cold tolerance triggered by soluble sugars: a multifaceted countermeasure. Front Plant Sci.

[36] Fowler S, Thomashow MF (2002). Arabidopsis transcriptome profiling indicates that multiple regulatory pathways are activated during cold acclimation in addition to the CBF cold response pathway. Plant Cell.

[37] Krasensky J, Jonak C (2012). Drought, salt, and temperature stress-induced metabolic rearrangements and regulatory networks. J Exp Bot.

[38] Castonguay Y, Nadeau P (1998). Enzymatic control of soluble carbohydrate accumulation in cold-acclimated crowns of alfalfa. Crop Sci.

[39] Cunningham SM, Nadeau P, Castonguay Y, Laberge S, Volenec JJ (2003). Raffinose and stachyose accumulation, galactinol synthase expression, and winter injury of contrasting alfalfa germplasms. Crop Sci.

[40] Uemura MG, Steponkus PL (2003). Modification of the intracellular sugar content alters the incidence of freeze-induced membrane lesions of protoplasts isolated from Arabidopsis thaliana leaves. Plant Cell Environ.

[41] Keunen E, Peshev D, Vangronsveld J, Van den Ende W, Cuypers A (2013). Plant sugars are crucial players in the oxidative challenge during abiotic stress: extending the traditional concept. Plant Cell Environ.

[42] Miura K, Furumoto T (2013). Cold signaling and cold response in plants. Int J Mol Sci.

[43] Irigoyen JJ, Emerich WD, Sanchez-Diaz M (1992). Water stress induced changes in concentrations of proline and total soluble sugars in nodulated alfalfa (Medkago sativa) plant. Physiol Plantarum.

[44] Cirousse C, Bournoville R, Bonnemain JL (1996). Water deficit-induced changes in concentrations in proline and some other amino acids in the phloem sap of alfalfa. Plant Physiol.

[45] Mohapatra SS, Poole RJ, Dhindsa RS (1987). Cold acclimation, freezing resistance and protein synthesis in alfalfa (Medicago sativa L. cv. Saranac). J Exp Bot.

[46] Gray GR, Chauvin L, Sarhan F, Huner NPA (1997). Cold acclimation and freezing tolerance: a complex interaction of light and temperature. Plant Physiol.

[47] Uemura M, Joeseph RA, Steponkus PL (1995). Cold acclimation of Arabidopsis thaliana: effect on plasma membrane lipid composition and freeze-induced lesions. Plant Physiol.

[48] Murata N, Los DA (1997). Membrane fluidity and temperature perception. Plant Physiol.

[49] Welti R, Li W, Li M, Sang Y, Biesiada H, Zhou HE, Rajashekar CB, Williams TD, Wang X (2002). Profiling membrane lipids in plant stress responses: role of phospholipase Dα in freezing-induced lipid changes in Arabidopsis. J Biol Chem.

[50] Ejsing CS, Duchoslav E, Sampaio J, Simons K, Bonner R (2006). Automated identification and quantification of glycerophospholipid molecular species by multiple precursor ion scanning. Anal Chem.

[51] Fahy E, Subramaniam S, Brown HA, Glass CK (2005). A comprehensive classification system for lipids. Eur J Lipid Sci Technol.

[52] Cyril J, Powell GL, Duncan RR, Baird WV (2002). Changes in membrane polar lipid fatty acids of seashore Paspalum in response to low temperature exposure. Crop Sci.

[53] Sung DY, Kaplan F, Lee KJ, Guy CL (2003). Acquired tolerance to temperature extremes. Trends Plant Sci.

[54] Trinchant JC, Boscari A, Spennato G, Van de Sype G, Rudulier LD (2004). Proline betaine accumulation and metabolism in alfalfa plants under sodium chloride stress. Plant Physiol.

[55] Devi J, Sanwal SK, Koley TK, Mishra GP, Karmakar P (2019). Variations in the total phenolics and antioxidant activities among garden pea (Pisum sativum L.) genotypes differing for maturity duration, seed and flower traits and their association with the yield. Sci Hortic.

[56] Giovanni A, Stefano B, Lucia G, Francesco F, Alessio F, Massimiliano T (2011). The biosynthesis of flavonoids is enhanced similarly by UV radiation and root zone salinity in L. vulgare leaves. J Plant Physiol.

[57] Cao D, Li H, Yi J, Zhang J, Che H (2011). Antioxidant properties of the mung bean flavonoids on alleviating heat stress. PLoS One.

[58] Tiziana C, Ivana P, Goffredo P, Giuseppe RR, Angela RLP (2011). Expression analysis in response to low temperature stress in blood oranges: implication of the flavonoid biosynthetic pathway. Gene.

[59] Schulz E, Tohge T, Zuther E, Fernie A, Hincha D (2016). Flavonoids are determinants of freezing tolerance and cold acclimation in Arabidopsis thaliana. Sci Rep.

